# Investigating clinical pharmacokinetics of brivaracetam by using a pharmacokinetic modeling approach

**DOI:** 10.1038/s41598-024-63903-1

**Published:** 2024-06-11

**Authors:** Attia Qayyum, Ammara Zamir, Muhammad Fawad Rasool, Imran Imran, Tanveer Ahmad, Faleh Alqahtani

**Affiliations:** 1 Department of Pharmacology, Faculty of Pharmacy, Bahauddin Zakariya University, Multan, 60800 Pakistan; 2 Department of Pharmacy Practice, Faculty of Pharmacy, Bahauddin Zakariya University, Multan, 60800 Pakistan; 3 Instiitute for Advanced Biosciences (IAB), CNRS UMR5309, INSERM U1209, Grenoble Alpes University, 38700 La Tronche, France; 4 Department of Pharmacology and Toxicology, College of Pharmacy, King Saud University, 11451 Riyadh, Saudi Arabia

**Keywords:** Pharmacology, Clinical pharmacology, Pharmacokinetics

## Abstract

The development of technology and the processing speed of computing machines have facilitated the evaluation of advanced pharmacokinetic (PK) models, making modeling processes simple and faster. The present model aims to analyze the PK of brivaracetam (BRV) in healthy and diseased populations. A comprehensive literature review was conducted to incorporate the BRV plasma concentration data and its input parameters into PK-Sim software, leading to the creation of intravenous (IV) and oral models for both populations. The developed physiologically based pharmacokinetic (PBPK) model of BRV was then assessed using the visual predictive checks, mean observed/predicted ratios (R_obs_/_pre_), and average fold error for PK parameters including the maximum systemic concentration (C_max_), the area under the curve at time 0 to t (AUC_0–∞_), and drug clearance (CL). The PBPK model of BRV demonstrated that mean R_obs_/_pre_ ratios of the PK parameters remained within the acceptable limits when assessed against a twofold error margin. Furthermore, model predictions were carried out to assess how AUC_0–∞_ is affected following the administration of BRV in individuals with varying degrees of liver cirrhosis, ranging from different child–pugh (CP) scores like A, B, and C. Moreover, dose adjustments were recommended by considering the variations in C_max_ and CL in various kidney disease stages (mild to severe).

## Introduction

Chronic diseases may cause changes in drug pharmacokinetics (PK) by inducing various pathophysiological alterations in comorbid patients, thus potentially requiring adjustments in drug therapy^1^. The advancement of technology has tremendously facilitated the building of proficient PK models, thus simplifying and speeding the modeling and simulation procedures^2–4^. As a result, these methodologies have become crucial parts of the drug development toolkit, leading to an increase in their utilization^5^. Physiologically based pharmacokinetic (PBPK) models have significantly enhanced drug advancement in the pharmaceutical industry by reducing the time needed to achieve data about the new drug’s PK profiles^6^. The PBPK modeling may not only help in the development of new drug compounds but also innovative drug dosage forms, offering options to include drug-related in-vitro data^7^. The prevalence of PBPK modeling has increased its role in managing various drug-drug and drug-disease interactions^8^.

Epilepsy is one of the most common neurological diseases, affecting over 1% of the global population, and reflects underlying brain dysfunctions^9^. Despite proper medication, more than one-third of patients have uncontrolled epilepsy^9^. In epileptic patients, combination or adjuvant therapy is the basis of treatment therefore, in such cases, it will be beneficial to create a PBPK model for predicting the alterations in its ADME^10^.

Brivaracetam (BRV) is an antiepileptic medicine used for status epilepticus and focal seizures^11^. It is present in both formulations oral and intravenous (IV). Its precise mode of action is uncertain, however, its anticonvulsant effects in the brain are attributed because of its higher affinity for synaptic vesicle protein (SV2A)^12^. It is thought to play an important function in neurotransmission regulation by inducing vesicle fusion and maintaining a reserve of secretory vesicles^12^. The plasma protein binding (PPB) of BRV with albumin is < 20% and plasma clearance is 3.4 L/h^12–15^. BRV is metabolized by the CYP2C19 enzyme with a substrate concentration at half of the maximum velocity (K_m_), lipophilicity (Log P), and fraction unbound (f_u_) values of 71.20 μM, 1.04, and 0.83, respectively^16^. Due to the involvement of the CYP2C19 enzyme, changes in hepatic or renal function may worsen the condition of patients which may require considerable monitoring. To configure the model, data of BRV under varying conditions, including both healthy and diseased states, was collected from previously published literature.

Liver cirrhosis is a clinical outcome of many liver diseases and is defined by fibrosis of tissues and the transformation of regular hepatic functions into structurally irregular nodules^17^. The changes in values of plasma protein scale factor (albumin), hematocrit, glomerular filtration rate (GFR), blood flow rate, and organ volumes are documented in the already published articles^16,18^ that are 0.92, 0.4205, 14.56, 24.01, and 1.734 respectively. Chronic kidney disease (CKD) is defined by abnormalities in the structure or function of kidneys, as well as a decrease in the estimated glomerular filtration rate (eGFR)^19^. The alterations in hematocrit, albumin levels, gastric emptying time, and small intestinal transit time are implemented as per standards outlined in previously reported literature^16,20^. Both liver cirrhosis and CKD may cause many pathophysiological alterations therefore, the incorporation of their parameters in the model development may help in the BRV dose optimization. The predictions based on this PBPK model may help clinical trial design in CKD and liver cirrhosis, as well as determining appropriate drug dosage based on disease severity (mild, moderate, and severe)^16,18,19^.

There has been only one published model on BRV in previous literature regarding the PK of BRV and its drug interaction with rifampin, a potent CYP2C19 inducer^16^. Therefore, the current study is focused on evaluating and developing a PBPK model for predicting BRV behavior in liver and kidney diseases, using a strategic model-building approach. Furthermore, the development of a drug-disease model may assist researchers in understanding the fundamental alterations in the PK parameters of BRV. The main purpose of the existing study is the establishment of a PBPK model in liver cirrhosis and CKD that may provide a reference for clinicians in the future to tailor doses in diseased subjects and thus promote rationalized drug administration.

## Methods

### Literature screening and search strategy

The relevant articles were retrieved after a comprehensive search from the Google Scholar and PubMed databases after BRV oral and IV routes of administration with the relevant data having concentration–time profiles in healthy and diseased subjects. The ultimate choice was determined by the comprehensive availability of data concerning weight, age, gender, and dose of BRV. In the case of healthy individuals, 5 oral studies and 1 IV study were included. Furthermore, 2 studies having drug concentration–time profiles for liver cirrhosis and CKD subjects were utilized for the development of the diseased model (Table 1). To carry out the process of extracting data, the Graph Digitizer (GetData version 2.26 software) was utilized to convert each graph from the included publications into digital form, facilitating model assessment and development.

**Table 1:** Study characteristics used for model development of brivaracetam.

Serial *#*	Study	N	Population	Gender	Age (years)	Weight (kg)	Dose (mg)	Route	Frequency
Healthy IV study									
1	Stockis et al.^21^	25	H	M/F	18–55	M ≥ 50 F ≥ 45	100	IV bolus	Single dose
Healthy oral studies									
1	Stockis et al.^21^	25	H	M/F	18–55	M ≥ 50 F ≥ 45	10, 50, 75, 100	Oral	OD
2	Stockis et al.^22^	14	H	M	18–55	≥ 50	200	Oral	OD
3	Rolan et al.^13^	35	H	M	18–55	≥ 50	10, 20, 40, 80, 150, 300, 600, 1000, 1400	Oral	OD
4	Stockis et al.^23^	42	H	19 M, 7 F	38–72	70–80	100	Oral	OD
5	Stockis et al.^24^	80	H	M	20–40	N/M	2.5–100	Oral	Single dose
							2.5–50		Multiple doses
Diseased studies									
1	Sargentini‐Maier et al.^25^	18	CKD	M/F	32–62	70–80	200	Oral	OD
2	Stockis et al.^23^	42	Liver cirrhosis	19 M, 7 F	38–72	70–80	100	Oral	OD

*H* healthy, *N* number, *CKD* chronic kidney disease, *M* male, *F* female, *OD* once a day, *N/M* not mentioned.

### Modeling software

The whole-body PBPK population-based simulator, version 11- build 150 PK-Sim (Biophysics Bayer Technology services, 42096 Wuppertal, Germany),^26^ was used to develop and evaluate the BRV PBPK model in healthy control and diseased subjects.

### The concept for model development

The Open System Pharmacology Suite (OSP) has developed a commercial software PK-Sim, that features a user-friendly graphical interface built upon a variety of diverse building blocks. Drug data were then utilized to construct a range of building blocks within the software. Drug parameters specific to the PBPK model were determined using the literature-reported values.

### Strategy for model development

**Figure 1:**
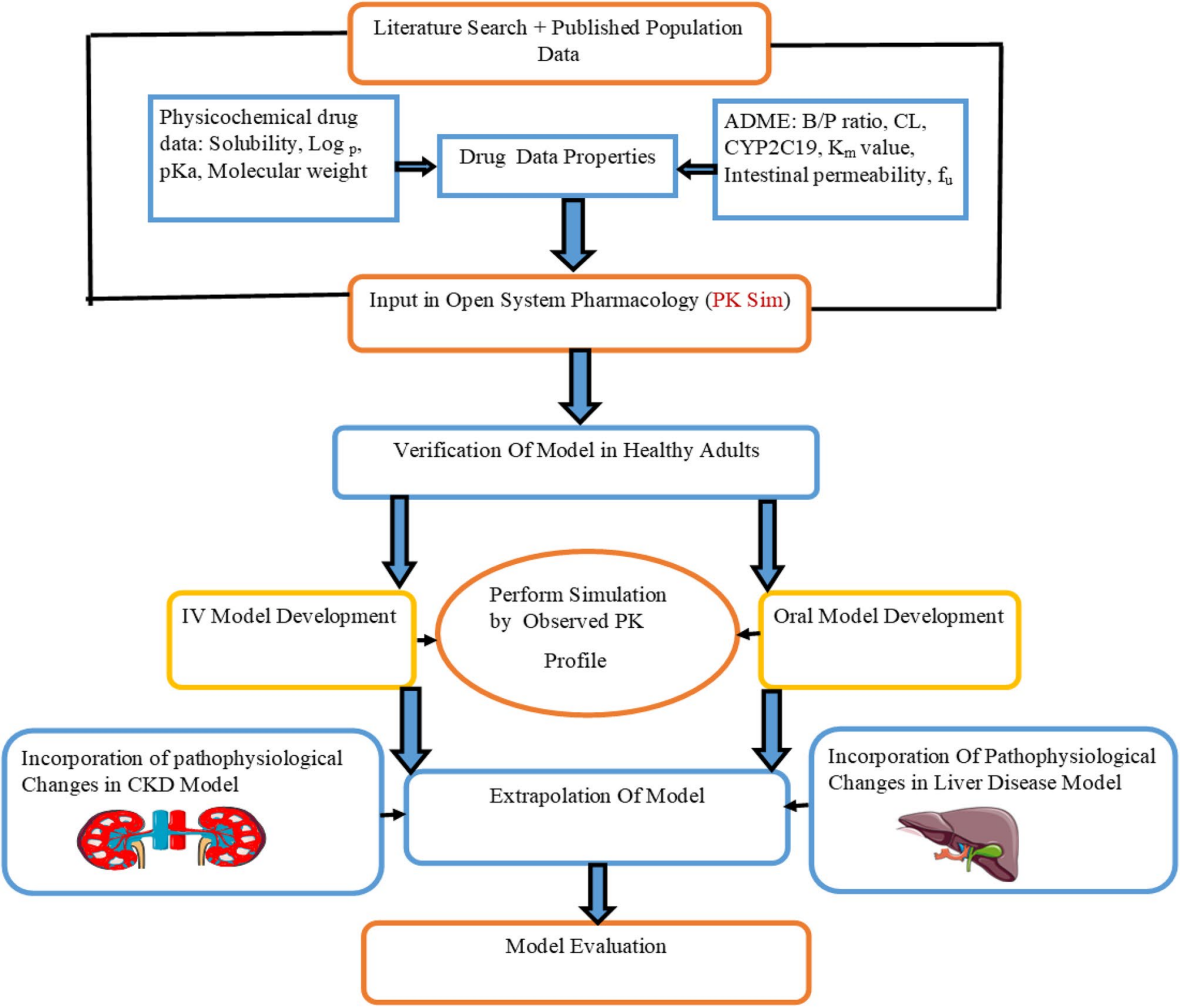
Brivaracetam PBPK model workflow presentation. *f*_*u*_ fraction unbound, *CYP* cytochrome P450, *pKa* dissociation constant, *Log P* lipophilicity, *K*_*m*_ substrate concentration at half-maximal velocity, *PBPK* physiologically based pharmacokinetic modeling, *B/P* ratio of blood to plasma, *ADME* absorption, distribution metabolism, and excretion, *IV* intravenous, *CL* clearance, Diagrammatic figure Part of the workflow was taken from the Servier Medical Art (SMART) that is licensed under 3.0 Unported License of a Creative Commons Attribution (https://creativecommons.org/licenses/by/3.0).

To commence the drug PBPK model development, the first step involved identifying various PK parameters. By using the established model-building techniques (involving the integration of physiological parameters and drug-specific properties to accurately predict drug distribution) the parameters for the selected PK profile and drug were integrated into the PK-Sim software^27,28^. Following that, sensitivity analysis was conducted for the model parameters such as Log P, water solubility, pKa, specific intestinal permeability, f_u_, and K_m_ value for CYP2C19. The model was developed and evaluated by using the already employed methodical model-building approach^29,30^. In this approach, the initial stage of model development focuses on predictions after IV administration in healthy adults, avoiding the complications linked to modeling the oral process of drug absorption. After successfully comparing the data related to IV, further predictions of the published PK data are formed for the oral drug administration, and a selection of factors impacting the process of drug absorption is determined. After the successful evaluation of the PBPK model in the healthy population, several pathological alterations associated with the diseases were integrated into the drug-disease model (Liver and CKD). Subsequently, the model was utilized for the prediction of drug PK in populations affected by specific diseases. The diagrammatic illustration for model development is depicted in Fig. 1.

### Model structure and parameterization

BRV is a drug with a molecular weight of 212.29 g/mol and a dissociation constant (pKa) of 7.07^16^. The PK-Sim compound file was created by using the values from the published article^16^. The main metabolic enzyme of BRV is CYP2C19, with a K_m_ value of 71.20 μM. The Rodgers and Rowland method for cellular permeability and partition coefficient was used in forecasting the model. However, this PK-Sim software includes an intrinsic feature that divides the gastrointestinal system into distinct compartments. Drug-related input parameters that were employed in development of BRV PBPK model are depicted in Table 2.

**Table 2:** Main drug-specific parameters and their integrated values in the PBPK model.

Physicochemical characteristics		
Input parameters	Values integrated into the model	References
Molecular weight (g/mol)	212.29	^16^
pKa	7.07	^16^
Solubility (mg/ml)	850	^16^
Log P	1.04	^9,16^
Absorption		
PPB	Albumin	^31,32^
Intestinal permeability	3.36 × 10^–6^	^16^
Distribution		
f_u_	0.79–0.83**	^9,16^
Partition coefficients	Rodgers and Rowland	^16^
Metabolism and elimination		
K_m_ CYP2C19 (μM)	71.20	^16^
K_cat_ CYP2C19	0.81	^16^
Renal CL (ml/min/kg)	0.06	^16^

*Log P* Lipophilicity, *pKa* dissociation constant, *f*_*u*_ fraction unbound, *PPB* plasma protein binding, *K*_*m*_ substrate concentration at maximal half velocity.

**The value integrated into the model was 0.61 based on a visual productive check.

### Diseased PBPK model structure

#### Chronic kidney disease (CKD)

CKD is classified into different stages according to eGFR (mild, moderate, and severe)^19^. Different disease-related physiological changes occur in CKD, such as time for gastric emptying, hematocrit, plasma protein (albumin), and transit time of small intestine that lead to alterations in the drug ADME^16,20^. The GFR was incorporated into the model for the moderate CKD profile (45 ml/min/1.73 m^2^) and severe CKD profile (20 ml/min/1.73 m^2^). The whole set of changes in different parameters like gastric emptying transit time, small intestine transit time, hematocrit, albumin, and GFR were then incorporated into the various populations established within the PK-Sim program. Therefore, all observed data were compared with the predicted CKD profiles for further assessment of the model. After creating a simulated prediction, the data is transferred from PK-Sim to the Graph Pad Prism analyzer for a comprehensive comparison of the relevant PK parameters like maximum systemic concentration (C_max_), the area under the plasma concentration–time curve (AUC_0–∞_), and CL.

#### Liver cirrhosis

Liver cirrhosis is a progressive condition marked by the deterioration of functional hepatocytes, with the formation of connective tissues and nodule formation within the liver. These alterations in both structure and function related to liver disease have a significant impact on drug PK^17,18,23,33,34^. The changes in the values of plasma protein scale factor (albumin), hematocrit, GFR, blood flow rate, and organ volumes are integrated into this disease model according to CP-A, CP-B, CP-C values as reported in the previously published articles^16,18,23^. Then these were compared with the healthy control and further graphical representation through Graph Pad Prism version 10 showed the visual representation of this comparison.

### Model validation and verification

A population of five hundred individuals was generated by using the computer-based models to represent all important and relevant PK profiles, as mentioned in the above-published articles. These variables include weight, age, dosage, route of administration, and formulations. The visual predictive check (VPC) approach was used for evaluating the PBPK model of BRV. The published reported data were compared with the predicted data, which encompassed the values from the 5th to 95th centile, the arithmetic mean, as well as a range of minimum and maximum values. By using the Microsoft Excel add-in program, PK Solver, a non-compartmental analysis (NCA) was conducted to calculate the PK parameters such as the area under the plasma (AUC_0–∞_), C_max,_ and CL, for both reported and predicted data. Following this, the ratio for observed and predicted (R_obs_/_pre_) mean and average fold error (AFE) for each PK variable (AUC_0–∞_, C_max_, and CL) were calculated by using Eqs. (1) and (2) (shown below). These calculations were performed for both healthy and subjects with liver cirrhosis.

1 $$\text{R }=\frac{Observed\, value\, of\, PK \,parameter}{Predicted \,value\, of \,PK\, parameter},$$

2 $$\text{AFE}= {10}^{\frac{\sum \text{log}(fold\, error)}{\text{N}}}.$$

## Results

### Healthy model evaluation after IV administration

**Figure 2:**
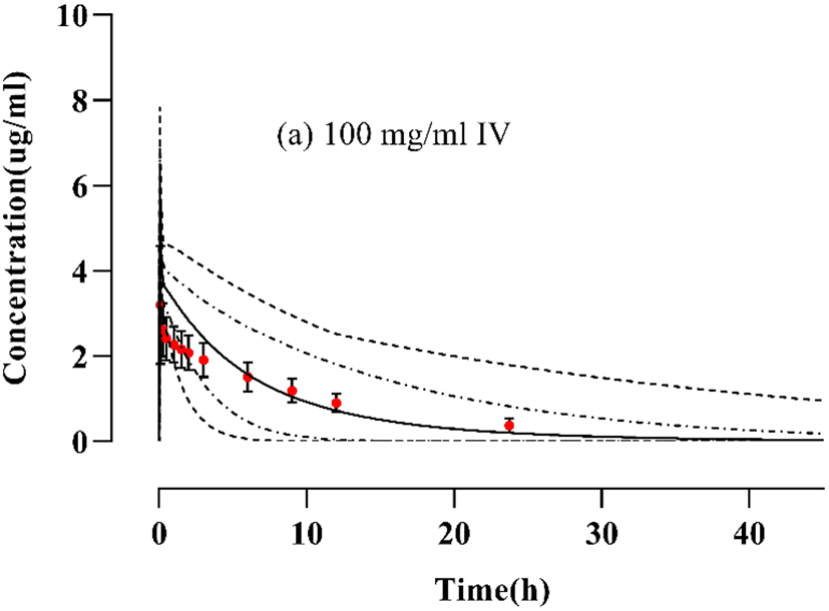
Concentration vs. time profiles comparison of observed and simulated profiles of Brivaracetam at (**a**) 100 mg/ml IV bolus^21^. The filled red coloured circle with SD (standard deviations) values describes the reported observed data values. The predicted data values are shown by solid lines, the maximum and minimum values as dashed lines, and the 5th and 95th percentiles as dotted lines.

For the establishment and development of the PBPK Model, after administration of 100 mg IV dose in healthy adults, the plasma concentration versus time profile of simulated and observed data was noted and compared. The reported data was confirmed by comparing with the simulated or predicted data after the IV administration of BRV 100 mg/ml dose, including the 5th–95th percentiles, arithmetic mean, minimum, and maximum (Fig. 2). After IV administration the PK parameter values for C_max_, AUC_0–∞,_ and CL were 0.93 µg/ml, 1.6 µg h/ml, and 0.62 ml/min/kg respectively (Table 3). Furthermore, to ascertain the validity of the PBPK model the R_obs_/R_pre_ of C_max_, AUC_0–∞_, and CL were calculated.

**Table 3:** R_obs/pre_ ratios of brivaracetam PK parameters for healthy population via oral route.

Administered dose (mg)	C_max_ (µg/ml)			AUC_0–∞_ (µg h/ml)			CL (ml/min/kg)			References
	Obs	Pre	R-value	Obs	Pre	R-value	Obs	Pre	R-value	
Intravenous administration profile										
100 mg/ml	3.796	4.065	0.93	31.421	19.524	1.6	3.182	6.121	0.62	21
Oral administration profile										
10	0.20	0.20	0.97	2.58	2.59	0.99	3.86	3.85	1.0	21
50	1.10	1.06	1.04	16.27	13.15	1.2	3.07	3.80	0.8	21
75	1.74	1.602	1.08	23.48	20.32	1.15	3.19	3.68	0.86	21
100	2.19	2.14	1.025	32.75	27.0	1.2	3.05	3.69	0.82	21
200	3.632	3.26	1.1	47.2	28.04	1.6	4.23	7.13	0.59	22
100	2.244	1.885	1.19	29.6	19.70	1.5	3.375	5.07	0.66	23
10	0.20	0.16	1.25	2.45	1.443	1.69	4.076	6.928	0.58	13
20	0.35	0.33	1.07	3.87	3.269	1.18	5.159	6.117	0.84	13
40	0.7554	0.665	1.13	9.322	6.884	1.35	4.290	5.810	0.73	13
80	1.888	1.339	1.4	17.83	13.8	1.29	4.085	5.794	0.77	13
150	3.336	2.529	1.3	42.77	26.78	1.59	3.506	5.599	0.62	13
300	6.285	5.169	1.2	76.53	55.99	1.36	3.919	5.357	0.731	13
600	13.971	10.64	1.3	152.20	125.87	1.20	3.941	4.766	0.82	13
1000	23.147	18.19	1.27	279.08	236.04	1.18	3.583	4.236	0.84	13
1400	34.50	26.04	1.32	417.79	372.2	1.12	3.350	3.760	0.89	13
2.5	0.067	0.038	1.7	0.876	0.90	0.97	2.85	2.77	1.02	24
10	0.34	0.22	1.5	3.542	3.63	0.975	2.82	2.22	1.27	24
25	0.76	0.52	1.46	7.306	8.9	0.82	3.42	2.90	1.17	24
50	1.263	1.0	1.2	17.51	17.27	1.01	2.85	2.79	1.02	24
100	2.09	2.09	1.0	31.85	30.30	1.05	3.13	3.3	0.94	24
2.5	0.03	0.05	0.6	26.31	25.90	1.01	0.09	0.07	1.3	24
10	0.14	0.22	0.63	54.51	53.51	1.02	3.8	3.85	0.98	24
50	0.72	0.61	1.18	153.09	152.9	1.0	3.81	3.85	0.98	24

*Obs* observed, *Pre* predicted, *C*_*max*_ maximum systemic concentration, *CL* clearance, *AUC*_*0–∞*_ area under the curve from time 0 to ∞, *H* healthy.

### Healthy model evaluation after oral administration

**Figure 3:**
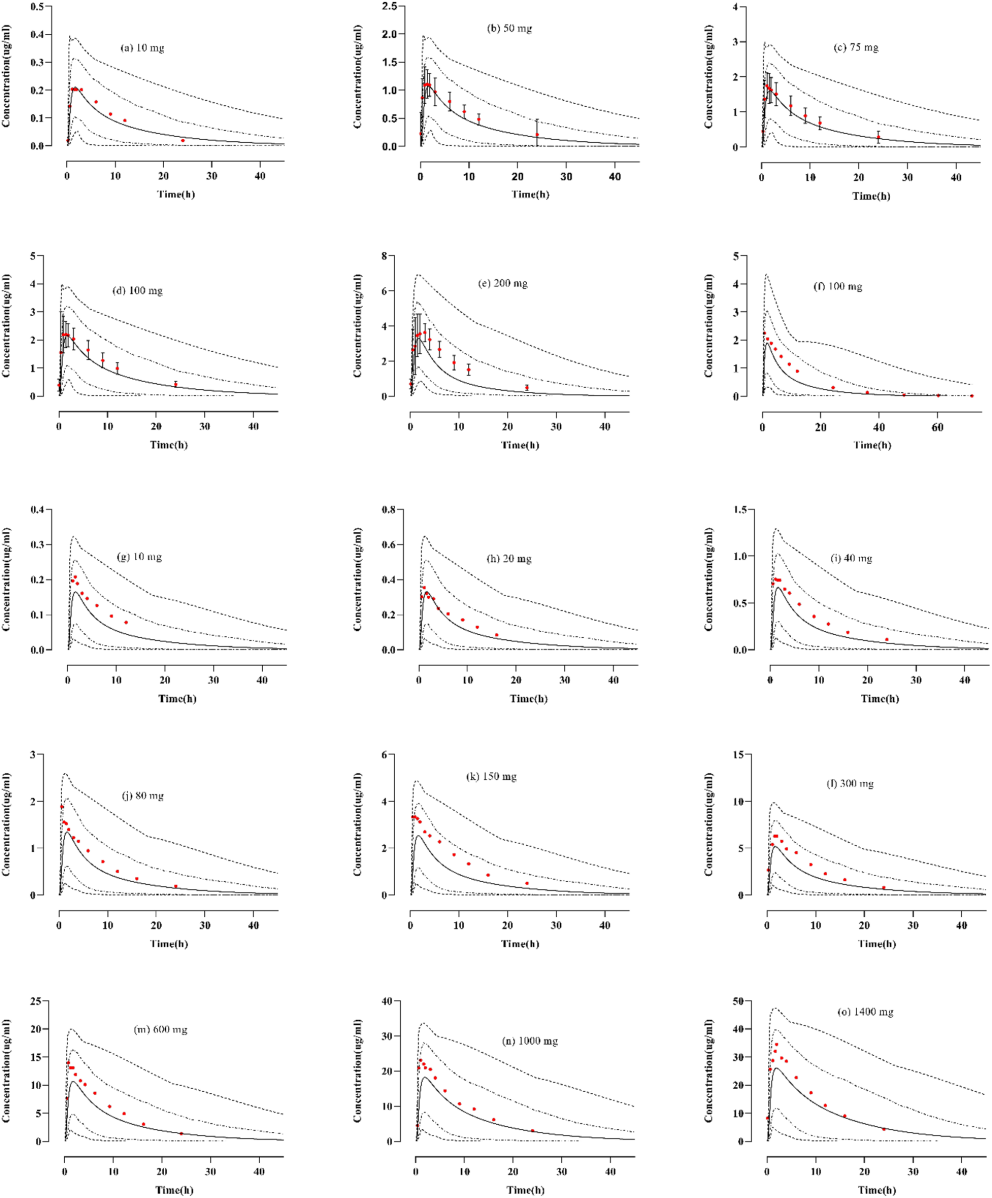
Brivaracetam concentration versus time profiles after oral doses of (**a**) 10 mg^21^, (**b**) 50 mg^21^, (**c**) 75 mg^21^, (**d**) 100 mg^21^, (**e**) 200 mg^22^, (**f**) 100 mg^23^, (**g**) 10 mg^13^, (**h**) 20 mg^13^, (**i**) 40 mg^13^, (**j**) 80 mg^13^, (**k**) 150 mg^13^, (**l**) 300 mg^13^, (**m**) 600 mg^13^, (**n**) 1000 mg^13^, (**o**) 1400 mg^13^. The coloured red circle explains the observed data values and standard deviation (SD where reported in the studies). The predicted data values are depicted by solid lines for simulated data, dashed lines for minimum and maximum values, and dotted lines for percentiles (5th–95th).

To evaluate the oral model, the reported data was compared with the predicted data, following different doses of BRV (10–1400 mg) by determining the concentration–time profiles, arithmetic mean or geometric mean, minimum, maximum, and 5th–95th percentile (Fig. 3). To confirm the precision of the PBPK model, AFE is calculated for PK parameters. The AFE values of C_max_, and AUC_0–∞_, for all oral doses, were 1.2, and 1.30 respectively, falling within a two-fold error range (Table 4) and the graphical representation of single and multiple oral doses are described in Supplementary Fig. S1.

**Table 4:** AFE computation for PK variables in healthy subjects.

PK parameters variables	AFE values
Healthy (oral)	
C_max_	1.2
AUC_0−t_	1.17
CL	0.88

*C*_*max*_ maximum systemic concentration, *CL* clearance, *AUC*_*0−∞*_ area under the curve at time 0 to ∞.

### Evaluation of model in diseased subjects

#### Liver cirrhosis

**Figure 4:**
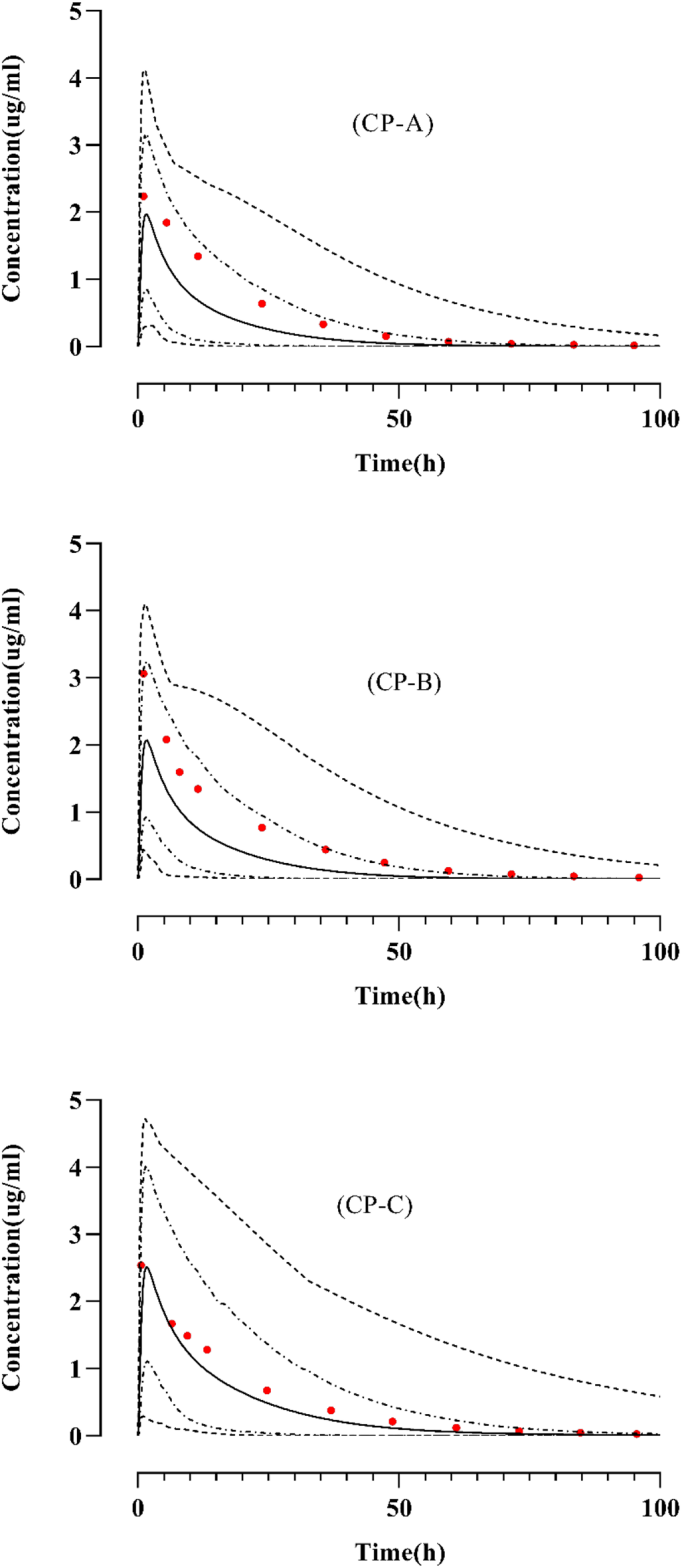
Concentration vs. time profiles comparison of observed and simulated profiles of brivaracetam (**A**) 100 mg^23^ CP-A, (**B**) 100 mg^23^ CP-B, (**C**) 100 mg^23^ CP-C. The reported data values are represented by red dots, predicted values as solidified lines, minimum and maximum values as dashed lines, and 5th and 95th percentiles as dotted lines. *CP* Child–Pugh.

For the evaluation of the model’s precision regarding liver cirrhosis, the observed data was aligned with the simulated systemic BRV concentration–time profiles after oral administration, demonstrating similarity with the arithmetic mean and 5th–95th percentile (Fig. 4). The mean R_obs_/R_pre_ ratio of C_max_ and CL are 1.433 and 0.9 respectively (Table 5). Moreover, the calculated AFE values were determined to fall within a two-fold error range, as illustrated in Table 6.

**Table 5:** R_obs/pre_ ratios of brivaracetam PK parameters for liver cirrhosis after oral administration.

Dose (mg)	Population	C_max_ (µg/ml)			AUC_0–∞_ (µg h/ml)			CL (ml/min/kg)			Reference
		Obs	Pre	R-value	Obs	Pre	R-value	Obs	Pre	R-value	
100	CP-A	2.2369	1.840	1.2	44.51	43.0	1.0	2.398	2.312	1.03	^23^
	CP-B	3.062	1.916	1.5	42.50	39.12	1.1	2.207	2.510	0.9	
	CP-C	2.541	1.597	1.6	46.30	36.90	1.25	2.167	2.910	0.74	

*Obs* observed, *Pre* predicted, *C*_*max*_ maximum systemic concentration, *CL* clearance, *AUC*_*0–∞*_ area under the curve from time 0 to ∞, *CP* Child–Pugh.

**Table 6:** AFE Computation for the PK variables in liver cirrhosis disease.

PK parameters variables	AFE values
C_max_	1.43
AUC_0–∞_	1.1
CL	0.9

*C*_*max*_ maximum systemic concentration, *CL* Clearance, *AUC*_*0−∞*_ area under the curve at time 0 to ∞.

#### Chronic kidney disease

Following the administration of BRV 200 mg the PK parameter values were observed that were found to be comparable among the CKD population (moderate and severe). A noticeable comparison between AUC_0–∞_values with minimum and maximum ranges in healthy control to moderate and severe CKD populations was depicted with values of 26.97 (10.56–55.66), 34.39 (6.811–60.71), 31.23 (8.149–51.51) respectively. Moreover, the details are represented in Table 7.

**Table 7:** PK parameter in healthy and CKD populations.

PK parameters			
Populations	C_max_ (µg/ml)	AUC_0–∞_ (µg h/ml)	CL (ml/min/kg)
	Mean (range)	Mean (range)	Mean (range)
Healthy	0.4976 (0.3114–0.7082)	26.97 (10.56–55.66)	1.624 (0.5853–3.418)
Moderate	0.5873 (0.2529–0.8952)	34.39 (6.811–60.71)	1.430 (0.5430–4.951)
Severe	0.5871 (0.2132–0.7602)	31.23 (8.149–51.51)	1.889 (0.5418–5.578)

*CL* clearance, *C*_*max*_ maximum systemic concentration, *AUC*_*0−∞*_ area under the curve at time 0 to ∞.

## Discussion

This study has developed a PBPK model of BRV after IV and oral administration through a systematic method to predict its metabolism and distribution in healthy populations and those having liver cirrhosis, and CKD. This PBPK model was initially established and verified in healthy people by using previously published research publications^19,35^. The AFE values for C_max_ and CL after oral administration of BRV are 1.2 and 0.88 (twofold error range) respectively, indicating that the model has effectively encapsulated the drug’s ADME characteristics through the careful selection of appropriate input parameters of the drug. Following the establishment of the model in healthy individuals, the study extrapolated the assessment of ADME for BRV in populations with CKD and liver cirrhosis by incorporating various reported pathophysiological changes^29,36–39^. By considering this, a BRV model was developed to forecast its exposure in individuals with different stages of CKD and various degrees of liver cirrhosis, which could offer valuable insights for dosage adjustments^40^.

Through an extensive literature review, a study on single and multiple doses has been screened out which depicts their effect on individuals with different CYP2C19 genotypes extensive, intermediate, and poor metabolizer (EM, IM, PM) as well. The model included information on CYP2C19 EM, IM, and PM by incorporating the expressions while simulating this study. Unlike previous studies that performed simulations collectively on a profile, a different approach was utilized in this study because plasma concentration–time profiles were presented as one for all individuals with EM, IM, and PM genotypes in the study in contrast to separate ones. Due to this reason, the percentage of individuals was first calculated in each genotype according to the number defined in the study for different doses. After that, the population was created in PK Sim software separately to generate simulated data and then subsequently the simulated data was transferred for each genotype to GraphPad Prism to create graphical representations^24^.

Liver cirrhosis is linked to diverse pathophysiological alterations, such as diminished organ blood flows (hepatic and renal), lowered albumin concentration, decreased liver volume, and alterations in the number of liver enzymes that have an important role in exposure to hepatic clearance^40–42^. These pathophysiological alterations have been incorporated within the PK-Sim software for (CP, A–C) in the liver cirrhosis population^41^. In the liver disease model, following BRV oral administration, the AFE values for AUC_0−∞,_ and CL are 1.1 and 0.9 respectively, which are found within a two-fold error margin, showing that the disease model is accurately developed after incorporating PK and drug-related parameters accurately. The PBPK model for BRV in liver cirrhosis indicates a decrease in AUC_0–∞_ after oral administration in simulated values which may affect medication effectiveness emphasizing the need for precise dosing considerations to manage potential safety and efficacy concerns.

BRV disposition was thoroughly investigated by using the PK-Sim program for the development and assessment of this model. Furthermore, for oral administration, the mean observed AUC_0–∞_ value was 62.9 µg h/ml that corresponded to the simulated value of 54.2 µg h/ml^13,21,22^. The PBPK model demonstrates an effective assessment of BRV PK in healthy control and liver cirrhosis populations, with the R_obs/pre_ values for the PK parameters (AUC_0–∞_, CL, and C_max_) falling within the twofold error range.

BRV is categorized as a drug with 100% absorption after oral administration; consequently, changes in plasma protein concentrations may potentially influence its PK^14,31^. Previous research indicates that the alterations commonly manifest within the CKD population, and involve various factors such as the abundance of enzymes, notably CYP2C19, time for gastric emptying time, small intestine transit time, hematocrit, and albumin^16,18,20^. In the CKD study, observed data was not available in the reported article to be used in the model development within the PK Sim software, making it impossible to compare the PK time profile analysis data of observed and predicted values^25^, therefore, the predicted data was transferred directly into GraphPad Prism software. This was done to extrapolate the model by using a GraphPad Prism analyzer, enabling a precise and accurate assessment of PK parameters (AUC_0–∞_, C_max_, and CL) for predicting the ADME of BRV in individuals with CKD. The mean values with the minimum and maximum ranges depicted an increase in drug clearance in the case of severe renal impairment which suggests a decrease in the concentration of BRV; therefore dose monitoring is required in CKD patients.

Although the dose adjustment regarding special populations has already been defined in the drug label but describes only the changes in exposure in the case of special populations. This drug is metabolized by the CYP2C19 enzyme which is inhibited by various drugs such as omeprazole, fluoxetine etc. so in case of concomitant what-if scenarios, the exposure of BRV will be increased in case of hepatic and renal impairment which can be helpful for clinicians in the future while pursuing the personalized medicine.

The current study’s limitations in establishing and assessing the PBPK model involve the usage of the Get Data Graph Digitizer, which aids in converting graphical representations of BRV’s concentration–time profiles from different published clinical articles into a digital format. The model included a clinical evaluation of CKD that focused on individuals with mild to severe symptoms. The presented PBPK model was evaluated based on the reported mean PK data along with the standard deviation and this can be considered as a potential limitation. Another limitation of our research is a lack of reported observed CKD plasma concentration–time profiles due to which we couldn’t represent its comparison with the predicted data in the software. Furthermore, the study on the CYP2C19 genotype with multiple doses has not explained the data properly in graphs separately for all genotypes (EM, IM, PM), due to which we have simulated the graphs by dividing the individuals according to the percentages mentioned in the respective study in contrast to the ideal creation of simulations in the development of PBPK model.

## Conclusions

The PBPK model for BRV successfully predicts its metabolism and distribution in healthy and diseased populations. The PBPK diseased model for BRV underscores the need for cautious dosing in individuals with liver impairment due to substantial changes in the PK of BRV. Moreover, BRV undergoes metabolism by the CYP2C19 enzyme that is inhibited by various drugs so potential alterations in drug exposure and clearance can be predicted in special populations highlighting the significance of accurate dosing considerations for patients. Future considerations should emphasize personalized techniques to improve treatment success and reduce possible potential risks in this patient population. This improved model may assist clinical practitioners with valuable insights for dosage adjustments and therapeutic management in these patient populations.

### Supplementary Information

Supplementary Information.

## Data Availability

All data generated or analysed during this study are included in this published article and its Supplementary Information file.
