# Supplementary material for: Investigating clinical pharmacokinetics of brivaracetam by using a pharmacokinetic modeling approach
Source: Sci Rep. 2024 Jun 11;14:13357. doi: 10.1038/s41598-024-63903-1 (PMC11164859; doi:10.1038/s41598-024-63903-1)
Supplement: Supplementary file 1 — Supplementary Information. [file 41598_2024_63903_MOESM1_ESM.docx]

# Supplementary File

# Investigating Clinical Pharmacokinetics of Brivaracetam by Using a Pharmacokinetic Modeling Approach

Attia Qayyum^1^, Ammara Zamir^2^, Muhammad Fawad Rasool^2*^, Imran Imran^1^, Tanveer Ahmad^3^, Faleh Alqahtani^4^

^1^Department of Pharmacology, Faculty of Pharmacy, Bahauddin Zakariya University, 60800, Multan, Pakistan; [attia.cpeic@gmail.com](mailto:attia.cpeic@gmail.com) (A.Q), imran.ch@bzu.edu.pk (I.I)

^2^Department of Pharmacy Practice, Faculty of Pharmacy, Bahauddin Zakariya University, 60800, Multan, Pakistan; ammarazamir20@gmail.com (A.Z); fawadrasool@bzu.edu.pk (M.F.R)

^3^Instiitute for Advanced Biosciences (IAB). CNRS UMR5309, INSERM U1209. Grenoble Alpes University, La Tronche, 38700, France; [tanveer.ahmad@univ-grenoble-alpes.fr](mailto:tanveer.ahmad@univ-grenoble-alpes.fr) (T.A)

**^4^**Department of Pharmacology and Toxicology, College of Pharmacy, King Saud University, Riyadh 11451, Saudi Arabia; afaleh@ksu.edu.sa (F.A)

* Correspondence: fawadrasool@bzu.edu.pk (M.F.R), Afaleh@ksu.edu.sa (F.A)

**
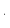

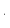
Supplementary Table 1**: **Physiological changes incorporated into the chronic kidney disease (CKD) and Liver cirrhosis.**

| **Physiological changes** | **Incorporated values** | | | |
| --- | --- | --- | --- | --- |
|  | **Moderate (CKD)** | **Severe (CKD)** | | |
| **Hematocrit** | 0.42 | 0.39 | | |
| **Plasma protein scale factor** | 0.99 | 0.90 | | |
| **Glomerular filtration rate (GFR)** | 45 mL/min/1.73 m^2^ | 20 mL/min/1.73 m^2^ | | |
| **Gastric emptying transit time** | 20.63 min | 24.38 min | | |
| **Small intestinal transit time** | 2.94 h | 2.94 h | | |
| **Liver Cirrhosis** | **A** | **B** | **C** |  |
| **Blood flow rates (ml/min/100g organ)** |  |  |  |  |
| Specific blood flow rate(bone) | 4.81 | 6.19 | 7.56 |  |
| Specific blood flow rate(brain) | 83.81 | 107.75 | 131.70 |  |
| Specific blood flow rate (fat) | 3.82 | 4.91 | 6.0 |  |
| Specific blood flow rate (gonads) | 14.11 | 18.14 | 22.17 |  |
| Specific blood flow rate (heart) | 112.98 | 145.26 | 177.54 |  |
| Specific blood flow rate(kidney) | 193.13 | 142.66 | 105.35 |  |
| Specific blood flow rate (large intestine) | 102.49 | 131.78 | 161.07 |  |
| Specific blood flow rate (liver) | 24.01 | 42.48 | 62.80 |  |
| Specific blood flow rate (muscle) | 5.99 | 7.69 | 9.41 |  |
| Specific blood flow rate (pancreas) | 58.39 | 75.94 | 91.77 |  |
| Specific blood flow rate (skin) | 15.14 | 19.46 | 23.79 |  |
| Specific blood flow rate (small intestine) | 145.93 | 187.62 | 229.32 |  |
| Specific blood flow rate (spleen) | 156.24 | 200.88 | 245.52 |  |
| Specific blood flow rate (stomach) | 62.77 | 80.71 | 98.64 |  |
| Duodenum (l/min) | 0.12 | 0.23 | 0.33 |  |
| Specific blood flow rate (upper jejunum) | 0.30 | 0.52 | 0.74 |  |
| Specific blood flow rate lower jejunum) | 0.30 | 0.52 | 0.74 |  |
| Specific blood flow rate upper ileum) | 0.33 | 0.54 | 0.80 |  |
| Specific blood flow rate (lower ileum) | 0.33 | 0.54 | 0.80 |  |
| Specific blood flow rate (cecum) | 0.05 | 0.09 | 0.11 |  |
| Specific blood flow rate (colon ascendens) | 0.09 | 0.14 | 0.22 |  |
| Specific blood flow rate (colon transversam) | 0.18 | 0.29 | 0.44 |  |
| Specific blood flow rate (colon descendens) | 0.14 | 225.0 | 0.33 |  |
| Specific blood flow rate (colon sigmoid) | 0.07 | 0.14 | 0.19 |  |
| Specific blood flow rate (rectum) | 0.04 | 0.05 | 0.08 |  |
| **Hematocrit** | 0.42 | 0.38 | 0.37 |  |
| **Plasma protein factor** | 0.92 | 0.76 | 0.59 |  |
| **GFR** | 14.56 | 12.16 | 11.57 |  |
| **Organ volume (l)** | 1.73 | 1.40 | 1.13 |  |

All these values are taken from the various studies ^1-4^

**Supplementary Figure 1:**


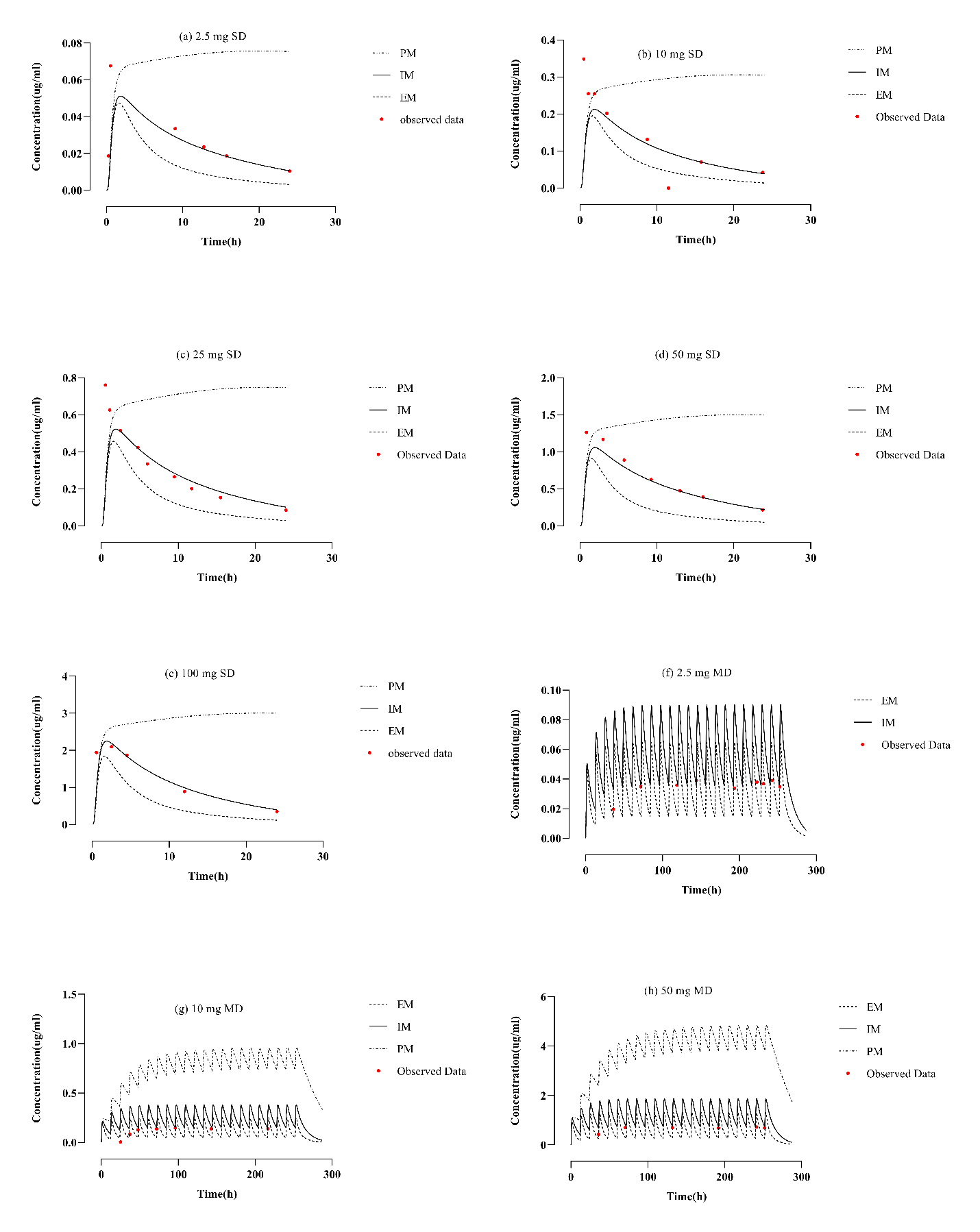
 **Figure 1:** Brivaracetam concentration versus time profiles after single and multiple oral doses of (a) 2.5 mg^5^ (b) 10 mg^5^ (c) 25 mg^5^ (d) 50 mg^5^ (e) 100 mg^5^ (f) 2.5 mg^5^ (g) 10 mg^5^ (h) 50 mg^5^. The red colored circle explains the observed data values. The predicted data values of intermediate metabolizer (IM) are depicted by solid lines, dashed lines for extensive metabolizer (EM), and dotted lines for poor metabolizer (PM).

Rerfernces

1 Malik, P. R. *et al.* A physiological approach to pharmacokinetics in chronic kidney disease. *The Journal of Clinical Pharmacology* **60**, S52-S62 (2020).

2 Rowland Yeo, K., Aarabi, M., Jamei, M. & Rostami-Hodjegan, A. Modeling and predicting drug pharmacokinetics in patients with renal impairment. *Expert review of clinical pharmacology* **4**, 261-274 (2011).

3 Johnson, T. N., Boussery, K., Rowland-Yeo, K., Tucker, G. T. & Rostami-Hodjegan, A. A semi-mechanistic model to predict the effects of liver cirrhosis on drug clearance. *Clinical pharmacokinetics* **49**, 189-206 (2010).

4 Edginton, A. N. & Willmann, S. Physiology-based simulations of a pathological condition: prediction of pharmacokinetics in patients with liver cirrhosis. *Clinical pharmacokinetics* **47**, 743-752 (2008).

5 Stockis, A., Watanabe, S., Rouits, E., Matsuguma, K. & Irie, S. Brivaracetam single and multiple rising oral dose study in healthy Japanese participants: influence of CYP2C19 genotype. *Drug Metabolism and Pharmacokinetics* **29**, 394-399 (2014).
